# Increased dysbindin-1B isoform expression in schizophrenia and its propensity in aggresome formation

**DOI:** 10.1038/celldisc.2015.32

**Published:** 2015-11-10

**Authors:** Yiliang Xu, Yuhui Sun, Haihong Ye, Li Zhu, Jianghong Liu, Xiaofeng Wu, Le Wang, Tingting He, Yan Shen, Jane Y Wu, Qi Xu

**Affiliations:** 1 National Laboratory of Medical Molecular Biology, Institute of Basic Medical Sciences, Chinese Academy of Medical Sciences and Peking Union Medical College, Tsinghua University, Beijing, China; 2 Department of Medical Genetics, School of Basic Medical Sciences, Capital Medical University, Beijing, China; 3 The State Key Laboratory of Brain and Cognitive Science, Institute of Biophysics, Chinese Academy of Sciences, Beijing, China; 4 Department of Neurology, Center of Genetic Medicine, Lurie Cancer Center, Northwestern University Feinberg School of Medicine, Chicago, IL, USA

**Keywords:** dysbindin-1, schizophrenia, protein aggregation, neurite outgrowth

## Abstract

Genetic variations in the human dysbindin-1 gene (*DTNBP1*) have been associated with schizophrenia. As a result of alternative splicing, the human *DTNBP1* gene generates at least three distinct protein isoforms, dysbindin-1A, -1B and -1C. Significant effort has focused on dysbindin-1A, an important player in multiple steps of neurodevelopment. However, the other isoforms, dysbindin-1B and dysbindin-1C have not been well characterized. Nor have been associated with human diseases. Here we report an increase in expression of *DTNBP1b* mRNA in patients with paranoid schizophrenia as compared with healthy controls. A single-nucleotide polymorphism located in intron 9, rs117610176, has been identified and associated with paranoid schizophrenia, and its C allele leads to an increase of *DTNBP1b* mRNA splicing. Our data show that different dysbindin splicing isoforms exhibit distinct subcellular distribution, suggesting their distinct functional activities. Dysbindin-1B forms aggresomes at the perinuclear region, whereas dysbindin-1A and -1C proteins exhibit diffused patterns in the cytoplasm. Dysbindin-1A interacts with dysbindin-1B, getting recruited to the aggresome structure when co-expressed with dysbindin-1B. Moreover, cortical neurons over-expressing dysbindin-1B show reduction in neurite outgrowth, suggesting that dysbindin-1B may interfere with dysbindin-1A function in a dominant-negative manner. Taken together, our study uncovers a previously unknown association of *DTNBP1b* expression with schizophrenia in addition to its distinct biochemical and functional properties.

## Introduction

Schizophrenia is one of the most devastating psychiatric disorders, affecting about 1% of the general population in their lifetime [1]. The association of gene encoding dysbindin-1 protein (*DTNBP1*) with schizophrenia has been repeatedly reported in multiple independent case-control studies [2–15]. Although no GWA studies have confirmed the association till now, reduced expression of dysbindin-1 mRNA and proteins has been observed in the brains of schizophrenia patients, including the dorsolateral prefrontal cortex [16] and the cerebral cortex [9]. Reduced dysbindin-1 protein levels have been found in a specific area of the hippocampus and immortalized lymphocytes of schizophrenia patients [17–19]. Moreover, sandy mice, which carry a dysbindin-1 null mutation (dysbindin-1^−/−^, Sdy), display electrophysiological deficits in auditory evoked response adaptation, prepulse inhibition and evoked γ-activity, similar to those electroencephalogram (EEG) patterns in patients with schizophrenia and schizophrenia-like behaviors such as locomotion and cognitional deficits [20–22]. Thus, *DTNBP1* is a strong candidate for schizophrenia susceptibility gene. Dysbindin-1 interacts with a number of proteins. For instance, as a component of BLOC-1 complex, dysbindin-1 is involved in intracellular membrane trafficking and organelle biogenesis [23–28]. Dysbindin-1 regulates neural glutamine release, microtubule assembly and activates PKA pathway by binding to snapin [29]. Dysbindin-1 binds to μ subunit of AP-3 complex to regulate exocytosis or soring of synaptic vesicle [30]. The function of dysbindin-1 also depends on its subcellular localization. At the presynaptic sites, dysbindin-1 is involved in the neurotransmitter release and retrograde, homeostatic modulation of neurotransmission [17, 31–35]. At postsynaptic sites, dysbindin-1 is required for receptor trafficking and endocytosis mediated by clathrin [36–42]. Dysbindin-1 contributes to neurite outgrowth, dendritic spine formation and neural differentiation [43–46].

**Figure 1:**
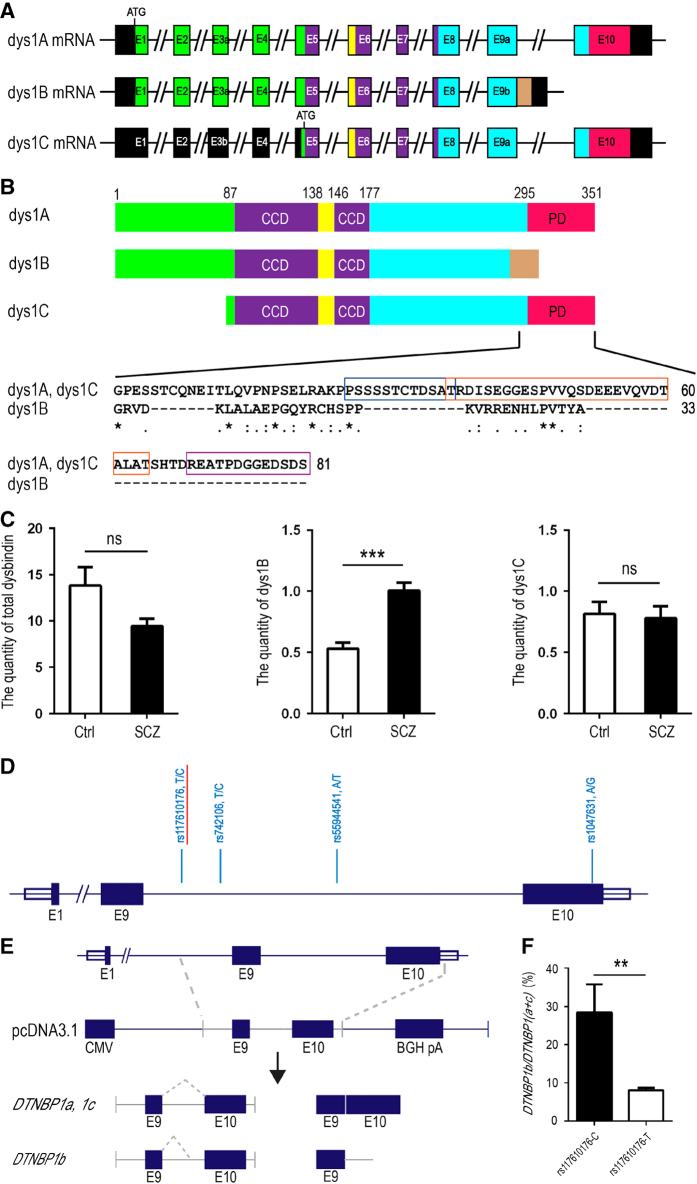
Differential expression of dysbindin-1 splicing isoforms in SCZ patients and *in vitro*. (**A** and **B**) Schematic illustrations of three major isoforms of dysbindin-1 mRNAs and their encoding proteins: dysbindin-1A (dys1A), dysbindin-1B (dys1B) and dysbindin-1C (dys1C). (**C**) The relative quantity of three dysbindin-1 isoforms of mRNA in the peripheral blood leukocytes in the controls (*n*=98) and patients with paranoid schizophrenia (*n*=98), as detected by quantitative PCR (qPCR). (**D**) A schematic illustration of 4 SNPs identified in the intron 9 and exon 10 of *DTNBP1* gene by preliminary genotyping. (**E**) A diagram depicting the *DTNBP1* minigene construct containing exon 9, intron 9 and exon 10. (**F**) The relative quantity of *DTNBP1b* and *DTNBP1(a+c)* mRNA in COS7 cells (*n*=6), as detected by qPCR. Data are presented as mean±s.e.m. **P<0.01, ***P<0.001. CCD, coiled-coil domain; PD, PEST domain.

Alternative splicing of the dysbindin-1 pre-mRNA mainly generates three transcripts, *DTNBP1a*, *1b* and *1c*, encoding dysbindin-1A, -1B and -1C proteins, respectively [47, 48], as depicted in Figure 1. Dysbindin-1A is the major isoform expressed in the central nervous system contributing to ~79% of the total dysbindin-1 proteins [48]. The carboxyl-termini of dysbindin-1A and -1C contain a domain rich in proline (P), glutamic acid (E), serine (S) and threonine (T) (PEST domain), a signature for protein degradation via the proteasome pathway or calpain proteolysis [49–51]. As a result of alternative splicing, dysbindin-1B does not contain the PEST domain [47] (Figure 1B). Dysbindin-1C is translated from an alternative start codon in exon 5, resulting in a peptide lacking the N-terminal 81 amino acids (Figure 1A and B). Recently, isoform-specific reduction in dysbindin-1 expression has been observed in different brain regions of schizophrenia patients [47, 48]. However, most genetic analyzes and functional studies on *DTNBP1* have focused on dysbindin-1A, whereas the functional role of other dysbindin isoforms in schizophrenia remains unclear.

Protein aggregation has an important role in the pathogenesis of a range of disorders affecting the central nervous system, including Alzheimer’s, Parkinson’s and Huntington’s diseases [52–55]. A recent study suggested that the protein encoded by the disrupted-in-schizophrenia 1 gene (*DISC1*) formed aggresomes when overexpressed in neurons [56]. Aggresomes are cytoplasmic 'inclusion bodies' at the microtubule-organizing center formed in response to discrete protein aggregates produced by misfolded proteins or excessive proteins [54, 55, 57]. Abnormal protein expression, degradation and subsequent aggresome formation may be involved in the pathogenesis of schizophrenia.

In this study, we have examined the differences in expression of all three dysbindin-1 mRNA isoforms between patients with paranoid schizophrenia and healthy control subjects, dysbindin-1B mRNA is elevated among the patients. Moreover, we have identified a single-nucleotide polymorphism (SNP) in the *DTNBP1* gene that is associated with schizophrenia and results in increased expression of dysbidnin-1B mRNA. Dysbindin-1B forms aggresomes both *in vitro* and *in vivo*. We also investigated the effect of dysbindin-1B on neurite outgrowth in cultured cortical neurons. Our study indicates that different dysbindin-1 isoforms have distinct functions in neural development and the disruption of the balance among different dysbindin-1 splicing isoforms may contribute to the pathogenesis of schizophrenia.

## Results

### Increased *DTNBP1b* mRNA expression in patients with paranoid schizophrenia

To examine the expression of three different isoforms, dysbindin-1A, -1B and -1C (Figure 1A and B), the total mRNA was extracted from peripheral blood leukocytes of healthy control and patients with paranoid schizophrenia. Quantitative real-time PCR (q-PCR) were performed using three pairs of primers specific for total dysbindin-1 mRNA, dysbindin-1B only and dysbindin-1C only, respectively (Supplementary Table S1). Consistent with a previous report [19], total *DTNBP1* transcripts were decreased in paranoid schizophrenic patients although the change was not statistically significant (Figure 1C, left panel). The *DTNBP1c* transcript level was not significantly different between patients and control, either (Figure 1C, right panel). However, the level of *DTNBP1b* mRNA was significantly higher in the patient group (1.00±0.07) than the control group (0.53±0.05; Figure 1B, middle panel, *P*<0.001). These results suggest that the expression of *DTNBP1b* mRNA was increased in patients with paranoid schizophrenia.

### Allelic and genotypic association of a SNP in intron 9 of the *DTNBP1* gene with paranoid schizophrenia

Dysbindin-1B is different from dysbindin-1A and -1C in exons 9 and 10 (Figure 1A). To identify the SNPs that may influence splicing of exon 9 and 10, a 2 071-bp fragment over exons 9 and 10, was amplified by PCR in 20 paranoid schizophrenic patients and 20 healthy control subjects, followed by bidirectional sequencing. However, only 4 out of 40 known SNPs in the NCBI database were confirmed in our samples, in which rs1047631 was excluded from further analysis due to a MAF of <5% (Figure 1D). The remaining three SNPs were genotyped in an expanded independent cohort (containing 500 healthy subject controls and 500 patients) and were verified to be in Hardy–Weinberg equilibrium in both case and control groups. Of these three SNPs, an allelic association with the disease was only detected for rs117610176-C allele located in intron 9 (*P*=6.78e-005, corrected *P*=0.0003; Table 1). Genotypic association was also detected for this SNP (*P*=3.22e-005, corrected *P*=0.0003; Table 2).

### Effect of rs117610176 SNP on alternative splicing of the *DTNBP1* gene

The location of rs117610176 SNP in intron 9 of the *DTNBP1* gene suggests a possible impact on alternative splicing of this gene. To test this hypothesis, we prepared the *DTNBP1* minigene constructs covering the sequence with 198-bp upstream region of exon 9, exon 9, intron 9 through exon 10 (Figure 1E) containing either the T or C allele of rs117610176 of the corresponding position in intron 9. These two constructs were transfected into COS7 cells, respectively. After 48 h, total mRNA was extracted and q-PCR was performed to compare the amounts of *DTNBP1b* relative to combined *DTNBP1a* and *DTNBP1c* (Supplementary Table S2). Interestingly, the level of *DTNBP1b* mRNA was significantly higher in the C allele-transfected COS7 cells than that of the T allele (*n*=6, *P*=0.0022) (Figure 1F), indicating that the rs117610176-C allele increases the splicing of *DTNBP1b* mRNA. Considering its association with paranoid schizophrenia, the rs117610176-C allele may contribute to the increased expression of *DTNBP1b* at mRNA level observed in these patients.

### Overexpression of dysbindin-1B and aggresome formation

**Figure 2:**
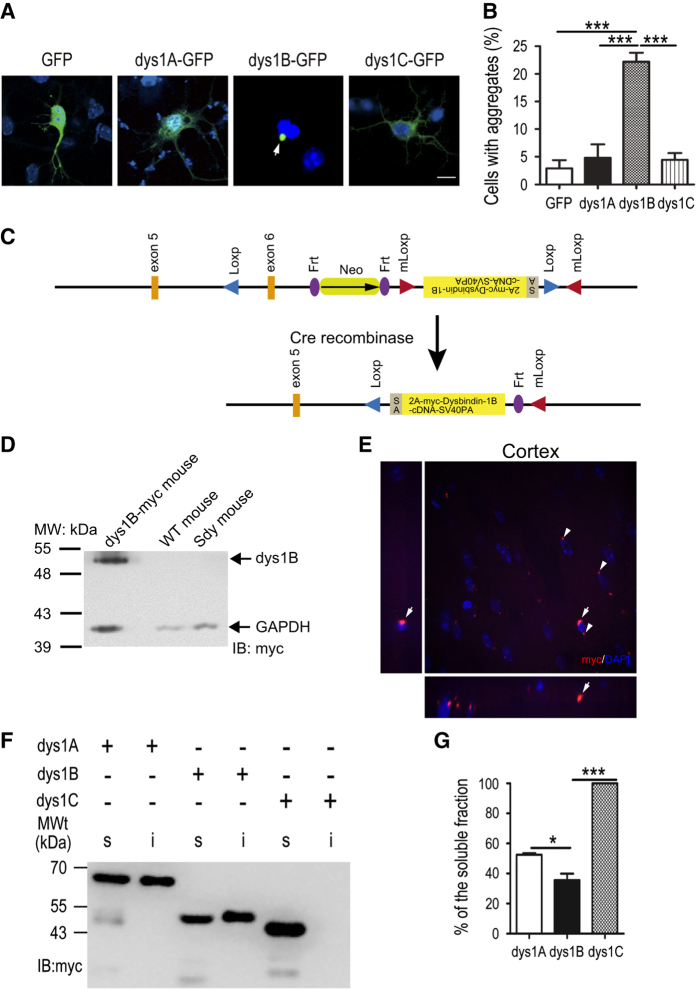
Differences in the subcellular distribution and solubility of dysbindin-1 isoforms and endogenous expression of human dysbindin-1B aggregates in mouse brain. (**A**) GFP-tagged dysbindin-1B forms aggregates in primary cultured cortical neurons (arrow). (**B**) The proportion of aggregate-containing neurons among those expressing GFP-tagged dysbindin-1A, -1B and -1C shown in panel **E**. Scale bars, 20 μm (panels **A** and **C**) and 5 μm (panel **E**). (**C**) A schematic diagram of a double LoxP system of human dysbindin-1B transgenic mouse model. (**D**) Endogenous expression of human dysbindin-1B in transgenic mouse is verified by western blot. (**E**) Endogenous expression of human dysbindin-1B in dysbindin1B^+/−, CMV-Cre^ mouse formed aggregates in the cortex (arrows and arrowheads). Aggregate indicated by arrow is shown in Z-stack. Scale bars, 5 μm. (**F**) The solubility of myc-tagged dysbindin-1A, -1B and -1C in transfected HEK293 cells. Transfected cells were lysed with RIPA buffer. The same volume of soluble (s) and insoluble (i) fraction was subjected to immunoblotting with anti-myc antibody. (**G**) The proportion of soluble myc-tagged dysbindin-1A, -1B and -1C in the total lysates. Data are presented as mean±s.e.m. *P<0.05, ***P<0.001.GFP, green fluorescent protein.

The variation in protein sequences and differential changes of mRNA levels of dysbindin-1 isoforms in patients with paranoid schizophrenia (Figure 1) suggested that different dysbindin-1 isoforms might have distinct biological properties. To test this hypothesis, we examined the subcellular localization of these dysbindin-1 isoforms. The three dysbindin-1 isoforms were tagged with green fluorescent protein (GFP) and transfected into COS1 cells. Interestingly, dysbindin-1B exhibited different subcellular distributions from the other two isoforms (Supplementary Figure S1A). In ~27% of transfected COS1 cells, dysbindin-1B-GFP formed aggregates in the perinuclear region (Supplementary Figure S1A and S1B). However, such aggregates were observed in fewer than 6% of dysbindin-1A-GFP or dysbindin-1C-GFP transfected cells, similar to the percentage observed in GFP control-transfected cells (Supplementary Figure S1A and S1B). Consistently, dysbindin-1B-GFP was more likely to form aggregates than dysbindin-1A-GFP or dysbindin-1C-GFP in transfected HEK293 cells (Supplementary Figure S1E and S1F). To rule out the possible aggregative effect associated with the GFP tag, we transfected cells with myc-tagged dysbindin-1A, -1B and -1C, and found that around 42% of COS1 cells expressing dysbindin-1B-myc formed perinuclear aggregates whereas <5% of cells expressing dysbindin-1A-myc or dysbindin-1C-myc contained such aggregates (Supplementary Figure S1C and S1D), indicating that the aggregation of dysbindin-1B is not caused by the GFP tag. Similarly, there are significantly more perinuclear aggregate-forming cells in dysbindin-1B-GFP-transfected mouse cortical neurons than the other two isoforms (Figure 2A and B). Because dysbindin-1B is not expressed in the mouse brain, to study the property and functions of dysbindin-1B *in vivo*, we made an inducible mouse model that expresses human dysbindin-1B under the control of endogenous promoter of *Dtnbp1* in the presence of Cre recombinase (Figure 2C and D). Dysbindin-1B aggregates were observed in the cortex in these dysbinin-1B^+/−, CMV-Cre^ mice (Figure 2E).

**Figure 3:**
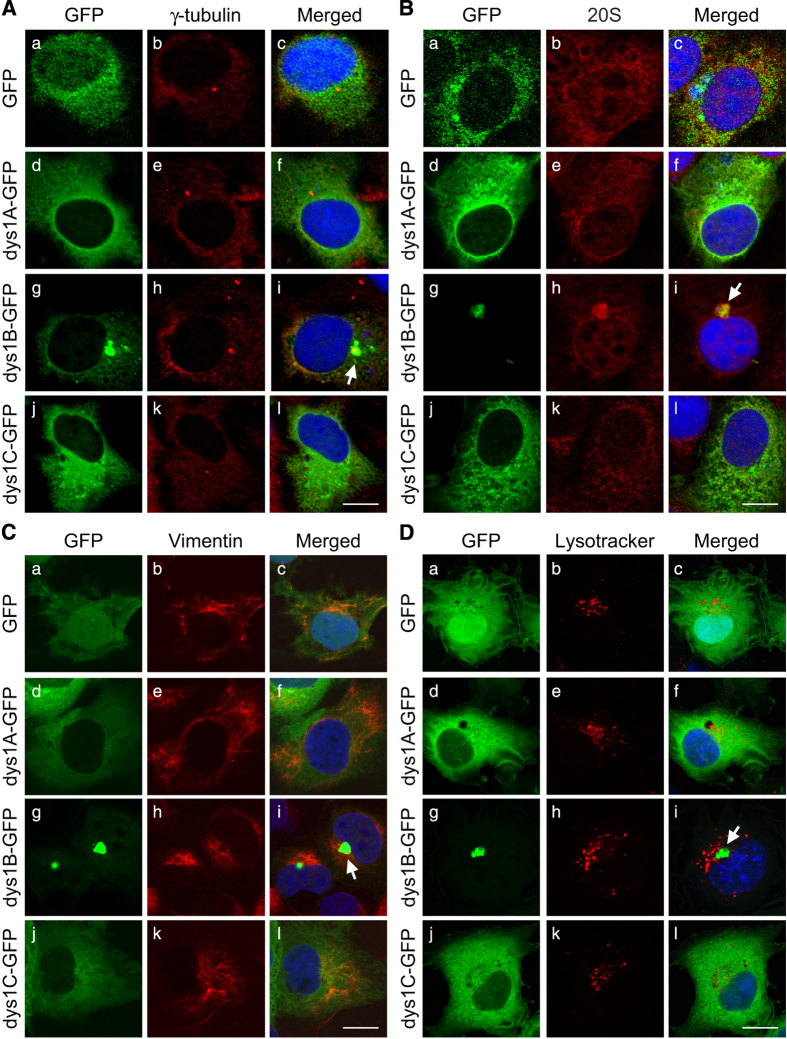
Colocalization of dysbindin-1B with aggresomal proteins. (**A**–**D**) COS1 cells were transfected with GFP-tagged control or dysbindin-1A, -1B and -1C (a, d, g, j of **A**–**D**; green) and immunostained with antibodies against γ-tubulin, 20S proteasomal subunit, or vimentin (b, e, h, k of **A**–**C**; red). The merged confocal images demonstrate colocalization of GFP-tagged dysbindin-1B with the two aggresome markers (c, f, i, and l of **A** and **B**; merged). The intermediate filament vimentin was surrounded with the aggregates (**i** of **C**). The arrows mark aggregates. (**D**) GFP-tagged dysbindin-1A, -1B or -1C expressing COS1 cells (a, d, g, j; green) were stained with a lysosomal marker, LysoTracker (b, e, h, k; red). Aggregates of GFP-tagged dysbindin-1B (arrow) were not colocalized with LysoTracker signals (c, f, i, l; merged). Scale bar, 20 μm.

The morphology and perinuclear distribution of aggregates formed in dysbindin-1B-expressing cells resembled those of aggresomes, the insoluble cytoplasmic 'inclusion bodies' formed around centrioles in response to the production of misfolded proteins or excessive protein expression [58]. A large portion of dysbindin-1B-myc protein (~61%) was detected in the RIPA buffer-insoluble fraction of cell lysates, whereas ~52% of dysbindin-1A-myc and almost 100% dysbindin-1C-myc were present in the RIPA buffer-soluble fractions (Figure 2F and G). We stained the dysbindin-1B-GFP-expressing cells with various aggresome markers, including γ-tubulin, 20S proteasome subunit and vimentin [59]. The aggregation of dysbindin-1B had a core positive for γ-tubulin, a marker for the centrioles and microtubule-organizing center (Figure 3A). These dysbindin-1B aggregates were colocalized with the 20S proteasome subunit (Figure 3B) and surrounded by the intermediate filament vimentin (Figure 3C), but not colocalized with lysosomes (Figure 3D). All these features of dysbindin-1B aggregates are consistent with the characteristics of aggresomes [59], indicating that expression of dysbindin-1B, but not dysbindin-1A or -1C, leads to increased formation of aggresomes. Moreover, interaction between soluble dysbindin-1B and DISC1 were observed (Supplementary Figure S5). Previous studies reported that DISC1 forms aggresomes in multiple cell lines and neurons [56, 60], raising the possibility that susceptibility protein aggresomes are involved in pathogenesis of schizophrenia.

### The soluble dysbindin-1B level is less sensitive to MG132 treatment than dysbindin-1A or -1C

**Figure 4:**
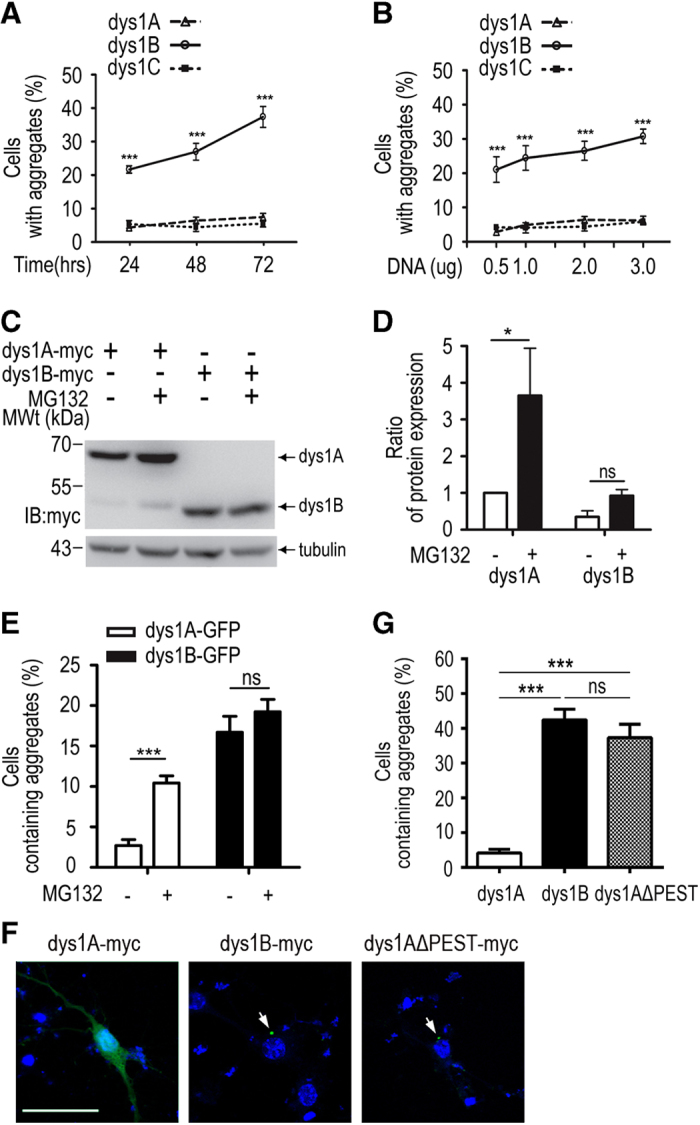
Different sensitivities of dysbindin-1A and dysbindin-1B to MG132 treatment and deletion of the PEST domain in dysbindin-1A results in aggregate formation. (**A**) The proportion of the COS1 cells containing aggregates 24, 48, and 72 h after transfection. (**B**) The proportion of the COS1 cells containing aggregates after transfection with 0.5–4 μg plasmids. (**C**) The levels of soluble myc-tagged dysbindin-1A and -1B in the absence or presence of MG132. (**D**) The ratio of soluble myc-tagged dysbindin-1A and -1B between MG132 treated and untreated cells. (**E**) The proportion of the MG132-treated COS1 cells containing aggregates. Scale bar, 20 μm. (**F**) Expression of dys1AΔPEST-myc deletion mutant in mouse cortical neurons leads to aggregate formation (arrow). (**G**) Quantification of the percentage of cells containing aggregates in mouse cortical neurons expressing myc-tagged dysbindin-1A, -1B and dysbindin-1AΔPEST. Scale bar, 40 μm. Data are presented as mean±s.e.m. *P<0.05,***P<0.001. ns, not significant.

Aggresomes are formed when some proteins are excessively produced, misfolded or fail to degrade. The proportion of cells containing aggregates was elevated when the expression level of dysbindin-1B was increased with prolonged transfection time or increased DNA quantity in COS1 cells (Figure 4A and B), suggesting that an increase in dysbindin-1B protein expression may lead to increased aggresome formation. However, increased transfection time or DNA quantity under the same conditions did not increase aggregation of dysbindin-1A or -1C (Figure 4A and B). This observation prompted us to investigate whether these isoforms were degraded through distinct pathways. Since dysbindin-1C only accounts for a small proportion of total dysbindin-1 in human brains as previously reported [48] and is highly soluble, our investigation mainly focused on dysbindin-1A and -1B. With MG132 treatment that inhibits the proteasome degradation pathway, the RIPA buffer-soluble fraction of dysbindin-1A was increased by 3.7-fold in transfected HEK293 cells whereas dysbindin-1B did not exhibit a significant change (Figure 4C and D). This result suggests that dysbindin-1A may be degraded via an MG132-sensitive proteasome degradation pathway whereas dysbindin-1B may use distinct MG132-insensitive degradation pathway. Interestingly, inhibition of dysbindin-1A degradation with MG132 also led to a 4-fold increase in the proportion of aggresome-forming COS1 cells but aggregation in dysbindin-1B-expressing cells was not significantly affected by MG132 treatment (Figure 4E).

The difference in dysbindin-1A and -1B degradation prompted us to investigate the underlying mechanism. Dysbindin-1A has a PEST domain containing three PEST sequences at the C terminus but dysbindin-1B lacks this PEST domain (Figure 1B). PEST sequences are signals for protein degradation via the proteasome pathway or calpain proteolysis [49–51]. To explore the role of PEST sequences in aggresome formation, we made a truncated form of dysbindin-1A (dys1AΔPEST-myc), in which the C-terminal PEST domain was removed. The proportion of aggregation-forming cells in dys1AΔPEST-myc transfected mouse cortical neurons was significantly higher than neurons transfected with the full-length dysbindin-1A-myc, and was similar to that in dysbindin-1B-myc transfected cells (Figure 4F and G). Similarly result was repeated in COS1 cells (Supplementary Figure S1C and 1D). Together, our results indicate that the lack of PEST sequence in dysbindin-1B may interfere with its proteasome-dependent degradation, leading to protein accumulation and aggresome formation.

### Dysbindin-1B interacts with dysbindin-1A and recruits it to aggresomes

**Figure 5:**
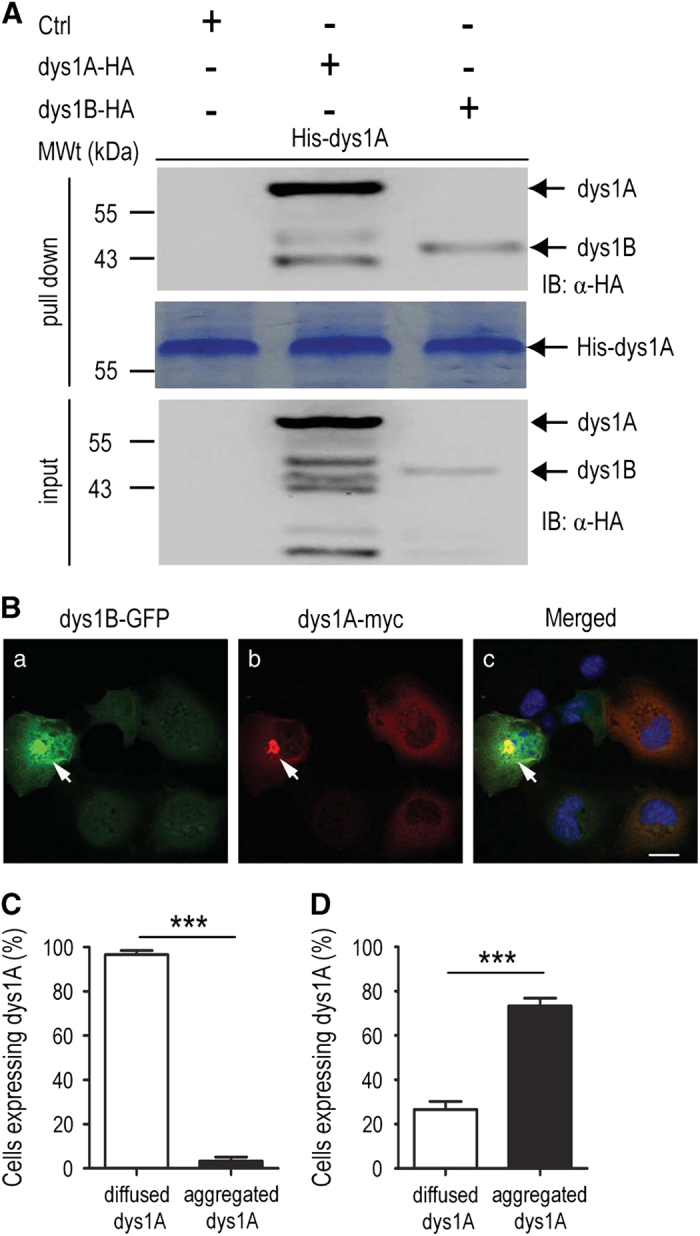
Analysis of the interaction between dysbindin-1A and dysbindin-1B. (**A**) His-tag pull-down assay. HA-tagged dysbindin-1A and -1B were detected in the proteins pulled down by purified His-dysbindin-1A. Coomassie blue staining showed the purified His-dysbindin-1A. (**B**) Immunostaining of cells co-transfected with myc-tagged dysbindin-1A and GFP-tagged dysbindin-1B. The arrow shows the diffused expression pattern of myc-tagged dysbindin-1A and GFP-tagged dysbindin-1B in co-transfected COS1 cells. The arrow marks the colocalization of myc-tagged dysbindin-1A with GFP-tagged dysbindin-1B in the dysbindin-1B aggregate-containing cells at the perinuclear region. (**C**) The proportion of cells expressing diffused or aggregated dysbindin-1A in co-transfected cells with diffused dysbindin-1B expression. (**D**) The proportion of cells expressing diffused or aggregated dysbindin-1A among cells containing dysbindin-1B aggregates. Data are presented as mean±s.e.m. ***P<0.001. Scale bar, 20 μm. GFP, green fluorescent protein.

Both dysbindin-1A and -1B contain coiled-coil domains that are involved in protein-protein interaction. A previous study showed that dysbindin-1A purified from the *E. coli* expression system was detected as oligomers by size exclusion chromatography (SEC) [56]. We performed crosslinking experiments in transfected cells and confirmed the oligomerization property of dysbindin-1A protein (Supplementary Figure S2). Consistently, HA-tagged dysbindin-1A interacted with purified His-tagged dysbindin-1A protein in the pull-down assay (Figure 5A). These results indicate that dysbindin-1A protein has homophilic binding property. Because dysbindin-1B expressed in *E. coli* was insoluble, we were unable to purify soluble dysbindin-1B protein for *in vitro* interaction experiments. To test whether dysbindin-1A interacted with dysbindin-1B, HEK293 cells were transfected with HA-tagged dysbindin-1B, and cell lysates were prepared and subsequently incubated with the purified His-dysbindin-1A protein in a pull-down assay. Dysbindin-1B was detected in the pull-down fraction (Figure 5A, lane 3), indicating that dysbindin-1B was capable of interacting with dysbindin-1A. Neither dysbindin-1A nor -1B was detected in the pull-down fractions in negative control samples using another unrelated protein, purified His-Smt3 (Supplementary Figure S3), demonstrating the specificity of the interaction between dysbindin-1A and -1B.

To test whether dysbindin-1B interacted with dysbindin-1A in cells, immunofluorescent microscopy was performed in COS1 cells co-transfected with dysbindin-1B-GFP and dysbindin-1A-myc. In cells with diffused dysbindin-1B-GFP in the cytoplasm, dysbindin-1A-myc also displayed a diffused distribution pattern (Figure 5B and C). However, ~75% of the cells containing dysbindin-1B-GFP aggresomes also showed dysbindin-1A-myc in the aggresomes (Figure 5B and D). After deleting the two coiled-coil domains both in dysbindin-1A and -1B, the interaction between these two proteins is eliminated (Supplementary Figure S4). These results suggest that dysbindin-1B may be able to recruit dysbindin-1A to aggresomes via heterophilic interaction of coiled-coil domains, thereby interfering with the normal distribution and function of dysbindin-1A protein.

### Inhibitory effect of dysbindin-1B on neurite outgrowth in primary culture of cortical neurons

**Figure 6:**
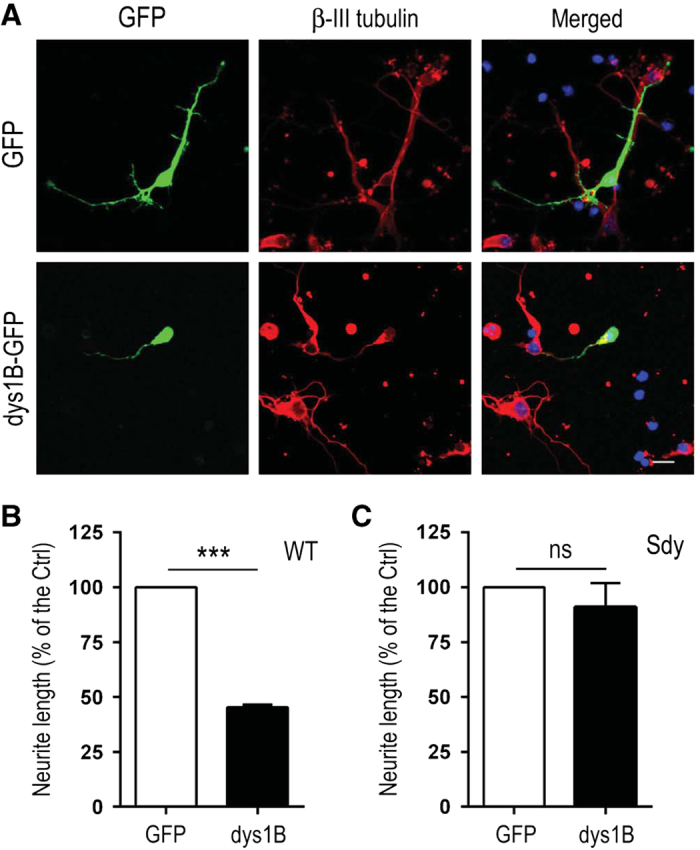
Inhibitory effect of dysbindin-1B on neurite outgrowth in cortical neurons. (**A**) Representative images of cortical neurons were obtained from wild-type E15.5 mice 48 h following transfection with GFP and GFP-tagged dysbindin-1B. (**B**) The relative average length of the longest neurites in neurons from wild-type mice expressing GFP and GFP-tagged dysbindin-1B. The GFP control was treated as 100%. (**C**) The relative average length of the longest neurites in neurons from sandy mice expressing GFP and GFP-tagged dysbindin-1B. The GFP control was treated as 100%. Data were collected from 3 independent experiments and presented as mean±s.e.m. ***P<0.001. ns, not significant. Scale bar, 20 μm. GFP, green fluorescent protein.

Differences in degradation and the subcellular localization of dysbindin-1A and -1B suggest that they may have distinct functional activities. It has been reported that mouse dysbindin-1 (as isoform 1A) facilitates neurite outgrowth by increasing p53 transcriptional activity in cultured cortical neurons [61]. We therefore examined the effect of dysbindin-1B on neurite outgrowth. Cortical neurons from embryonic stage 15.5 (E15.5) wild-type mice expressing endogenous dysbindin-1A or sandy mice lacking dysbindin-1 expression [38] were transfected with GFP control or dysbindin-1B (Figure 6). The longest neurites of individual neurons were measured. There were no significant difference in the neurite length among the control and dysbindin-1B-expressing neurons in neurons from the sandy mice lacking dysbindin (Figure 6C). However, in cortical neurons isolated from wild-type mice, expression of dysbindin-1B led to a significant decrease in the neurite length as compared with neurons transfected with the GFP control (Figure 6A and B). Besides dysbindin-1A, dysbindin-1B also recruit Snpain to aggreome in COS1 cells (Supplementary Figure S6B) via heterophilic binding (Supplementary Figure S6A). Together, these results suggest that expression of dysbindin-1B inhibits neurite outgrowth, probably through inhibition of the endogenous dysbindin-1A or its partners. Because *DTNBP1b* mRNA level was increased in patients with paranoid schizophrenia (Figure 1C), axonal growth and neural circuitry formation might be impaired in these patients.

## Discussion

Alternative pre-mRNA splicing is a major mechanism for genetic and proteomic diversity. Dysregulation of alternative splicing contributes to the pathogenesis of a wide range of human diseases. However, alternative splicing of genes associated with neuropsychiatric diseases and genetic variations/alterations affecting this crucial gene regulatory process remain underappreciated and insufficiently investigated.

*DTNBP1* is a major candidate gene for schizophrenia and has important roles in neural development. It encodes at least three isoforms of dysbindin-1 proteins in humans. Dysbindin-1 interacts with snapin, a component of Soluble NSF Attachment Protein Receptor complex, and regulates the levels of neurotransmitters, such as dopamine and glutamate, by controlling synaptic vesicle release at the presynaptic site [22, 29, 31, 32, 35, 62, 63]. At the postsynaptic site, dysbindin-1 is involved in controlling trafficking of the dopamine D_2_ receptor (D2), and in regulating cell surface expression and activity of the NMDA receptor [36, 38, 39, 41].

Dysbindin-1 also participates in neurodevelopmental processes. Knockdown of dysbindin-1 results in aberrant organization of the actin cytoskeleton in SH-SY5Y cells and in cultured hippocampal neurons [64]. It has also been reported that dysbindin-1 regulates dendritic development and promotes neurite outgrowth [43, 44, 61]. Dysbindin-1 regulates dendritic spine formation via interaction with Wiskott–Aldrich syndrome protein family verprolin-homologous protein 2 (WAVE2) and Abelson interacting protein-1 [45]. The complex containing dysbindin-1, biogenesis of lysosome related organelles complex 1, has a role in sorting cargoes from the cell body to the synapse, and deficiency in biogenesis of lysosome related organelles comple-1 disrupts neurite outgrowth [43, 65, 66]. Dysbindin-1 promotes neurite outgrowth possibly by recruiting necdin to the cytoplasm, attenuating the repressive effects of necdin and releasing the p53 transcriptional activity [67]. Primary cortical neurons from sandy mice display shorter neurites than those from wild-type mice [61].

Although a number of studies indicated that the *DNTBP1* gene was associated with schizophrenia and dysbindin-1 played important roles in neurotransmitter release and neural development, even in gene transcription regulation. Little is known about the mechanism by which the risk variants contribute to dysfunction of dysbindin-1 and pathogenesis of schizophrenia. As for different isoforms of dysbindin-1, more works on their function are needed. Here we report that increased expression of dysbindin-1B mRNA (*DTNBP1b*) is associated with paranoid schizophrenia. Dysbindin-1A and -1B proteins exhibit different solubility, in which dysbindin-1A is much more soluble than dysbindin-1B so that dysbindin-1B shows higher tendency to form aggresomes. Moreover, dysbindin-1B is able to recruit dysbindin-1A to the aggresome formation. Removal of the PEST domain at the C terminus of dysbindin-1A results in aggregate formation similar to dysbindin-1B overexpression. Dysbindin-1B inhibits neurite outgrowth, probably by heterophilic interaction with dysbindin-1A and impairs its function in neurons.

### The association of increased expression of *DTNBP1b* mRNA with paranoid schizophrenia

As a schizophrenia susceptibility gene, *DTNBP1* risk polymorphisms and haplotypes are associated with negative symptoms, cognition decline, early visual processing deficits and prefrontal brain function impairment in schizophrenia patients [68–74]. Imaging studies have shown that rs2619528 in *DTNBP1* is associated with reduced brain volume and regional cortical thickness in patients with schizophrenia [75]. Another *DTNBP1* risk haplotype (rs2619539-rs3213207-rs2619538) is also associated with reduced gray matter volumes in both the right dorsolateral prefrontal and left occipital cortex in schizophrenia [76]. The precise mechanisms by which these risk variations contribute to specific deficits in schizophrenia remain to be elucidated.

Reduced expression of *DTNBP1* mRNA and proteins has been found in schizophrenic brains, including multiple layers of the dorsolateral prefrontal cortex [16]. Subjects carrying *DTNBP1* risk haplotypes show reduced *DTNBP1* mRNA expression in their cerebral cortices [9]. In the hippocampal formation (HF), schizophrenia cases display presynaptic dysbindin-1 reductions specifically in the terminal fields of intrinsic, glutamatergic afferents of the subiculum, the hippocampus and especially the inner molecular layer of the dentate gyrus [17]. Reduced dysbindin-1 expression in the dentate granule and polymorph cells and in hippocampal CA3 neurons has also been reported [18]. The *DTNBP1* mRNA expression data from immortalized lymphocytes of schizophrenia appear to be rather inconsistent, but a number of studies suggest that reduced dysbindin-1 expression or function is likely to be associated with schizophrenia [19, 77].

Tang *et al,* reported that schizophrenia cases showed increased levels of *DTNBP1a* and *1b* mRNA but normal levels of *DTNBP1c* in the dorsolateral prefrontal corte, although western blotting analysis of the same tissue revealed significant reduction only in dysbindin-1C protein in schizophrenia patients [48]. The same group also reported that individuals with schizophrenia had alterations of synaptic dysbindin-1 isoforms in the posterior half of the superior temporal gyrus and HF, showing a reduction in synaptic dysbindin-1A but not dysbindin-1B and -1C in posterior half of the superior temporal gyrus. In the HF, however, schizophrenia cases displayed normal levels of synaptic dysbindin-1A but reductions in synaptic dysbindin-1B and -1C [47]. Recent studies showed that loss of dysbindin-1C result in loss of hilar mossy cells in HF due to impaired autophagy [78, 79], suggesting that different dysbindin-1 isoforms have distinct function in pathogenesis of schizophrenia.

In our study, increased expression of *DTNBP1b* mRNA was detected in the peripheral blood leukocytes of patients with paranoid schizophrenia (Figure 1C). The total dysbindin-1 mRNA was slightly reduced and no significant change in the dysbindin-1C mRNA was observed (Figure 1C). These results revealed the complexity in expression regulation of dysbindin-1 isoforms. Although we were not able to examine the isoform-specific expression pattern in brain tissue because of limited access to the brain samples, it is possible that altered expression of different dysbindin-1 isoforms could occur in different brain regions and during different developmental stages. Disruption in the balance between different dysbindin-1 isoforms may contribute to the pathogenesis of paranoid schizophrenia. However, whether rs117610176 and decreased *DTNBP1* mRNA are associated with other schizophrenia subtypes needs further investigations.

Because the nucleotide sequence downstream of the exon 9 is different among *DTNBP1a, DTNBP1c* and *DTNBP1b* mRNAs (Figure 1A and B), we further investigated the association of SNPs located in this region with paranoid schizophrenia at the genetic level. Interestingly, a SNP located in intron 9, rs117610176, showed strong association with paranoid schizophrenia (Tables 1 and 2). Our data suggest that the C allele of rs117610176 is likely to increase *DTNBP1b* expression (Figure 1F) that is consistent with the increase of *DTBNP1b* mRNA level in peripheral blood leukocytes of schizophrenic patients.

### Aggregated proteins in schizophrenia

Aberrantly expressed or misfolded proteins are very likely to form aggregates in the cytoplasm, leading to cellular damage [80, 81]. There are three major mechanisms involved in clearing the abnormal protein aggregates in cells, including molecular chaperon-assisted protein folding, ubiquitin–proteasome degradation and lysosome-autophagy pathways [55]. When these major degradation systems fail to degrade aggregated proteins efficiently, aggresomes may be formed around microtubule-organizing center or centriole as an additional cellular defense mechanism. PEST sequences enriched in proline (P), glutamic acid (E), serine (S) and threonine (T) are considered as a signature for rapid protein degradation [82]. Proteins with PEST sequences such as those playing regulatory roles in the physiological processes of cells are degraded through ubiquitin-dependent/independent proteasome pathways or proteolysis by calpain [49–51]. Our experiments show that increased expression of dysbindin-1B but not dysbindin-1A nor -1C leads to increased aggresome formation. Unlike dysbindin-1B, dysbindin-1A and -1C that contain a PEST domain at the C terminus are more soluble in RIPA buffer and more sensitive to proteasome inhibitor MG132 (Figure 2 and Figure 3). This suggests that insufficient degradation of dysbindin-1B resulting from lack of PEST domain may promote aggresome formation. Indeed, overexpression of dysbindin-1A can also lead to aggresome formation in cells when its PEST domains are removed (Figure 4A–E). Thus, increased dysbindin-1B expression and its lack of PEST sequences for efficient degradation may facilitate misfolded/unfolded proteins to form aggresomes in neurons.

Recent studies have suggested that protein aggregation may play a role in the pathogenesis of neuropsychiatric disorders. DISC1 protein forms aggresomes when overexpressed in neuroblastoma cells, COS cells and neurons [56, 60]. In neuroblastoma cells, overexpressed DISC1 is able to recruit homologous soluble C-terminal DISC1 fragment and heterologous dysbindin-1 to aggresomes, leading to their co-precipitation to the ionic detergent-insoluble fraction [56]. Consistently, increased ionic detergent-insoluble form of DISC1 protein and co-aggregation of DISC1 and dysbindin-1 are demonstrated in the postmortem brains of patients with affective disorders or schizophrenia as compared with control subjects [56, 83]. It has been proposed that aggresome formation may cause a loss of function of DISC1 by disrupting its interaction with partners, such as nuclear distribution element-like (NDEL1/NUDEL), or by impairing mitochondria transport and function, which may have a role in the pathogenesis of schizophrenia [60, 83]. The result that soluble dysbindin-1B interacts with DISC1 (Supplementary Figure S5) suggests that dysbindin-1B and DISC1 may co-exist in aggregates in schizophrenia patients. However, unlike Aβ and tau protein aggregates in Alzheimer’s disease, α-synuclein protein aggregates in Parkinson’s disease and polyglutamine protein aggregates in Huntington’s disease [52–54], the role of protein aggregation in the pathogenesis of schizophrenia remains unclear. The protein aggregation that results from imbalanced proteostasis may be more subtle in schizophrenia than classical neurodegenerative disorders [84]. Our results suggest that dysbindin-1A interacts with dysbindin-1B (Figure 4F and G). The balanced expression of dysbindin-1 isoforms may be important for normal function of neurons. Similar to DISC1, increased dysbindin-1B expression leads to aggresome formation and recruits dysbindin-1A to the aggresomes, which may disrupt function of dysbindin-1. Moreover, dysbindin-1B interacts with Snapin, another component of biogenesis of lysosome related organelles comple-1 complex *in vitro* (Supplementary Figure S6A), and recruits Snapin to agrresome in COS1 cells (Supplementary Figure S6B), implying dysbindin-1B interfer dysbindin-1A function by disrupting its interaction with partners such as DISC1 or snapin, or by impairing neurite outgrowth or neurotransmitter transport, which may have a role in the pathogenesis of schizophrenia.

### Neural developmental roles of dysbindin-1

Several studies suggest that impaired neurite outgrowth may be associated with schizophrenia. DISC1 regulates neurite outgrowth by interacting with NDEL1/NUDEL in differentiated PC12 cells and hippocampal neurons. This process is modulated by PKA phosphorylation [85–88]. The interaction between DISC1- and DISC1-binding zinc finger protein is necessary for pituitary adenylate cyclase-activating polypeptide-induced neurite outgrowth [89]. In addition, sandy mice that lack dysbindin-1 expression show schizophrenia-like phenotypes [20, 38, 90] and display deficits in neurite outgrowth and neuronal differentiation [46, 61]. *Dtnbp1* in mice only generates one splicing form equivalent to human dysbindin-1A [91], suggesting that dysbindin-1A is important for neurite outgrowth in the neurodevelopmental processes. Our results show that human dysbindin-1B suppresses neurite outgrowth only in the wild-type neurons expressing endogenous dysbindin-1A, whereas dysbindin-1B has no effect in sandy mice lacking dysbindin-1 protein (Figure 5B and C). The observation that dysbindin-1B recruits dysbindin-1A to the aggresome suggests that dysbindin-1B overexpression may interfere with the activity of endogenous dysbindin-1A and suppress neurite growth (Figure 5). Whether dysbindin-1B overexpression and formation of aggresomes compromise functions of other proteins critical for neurite outgrowth requires further investigation.

In summary, we show that rs117610176 is associated with paranoid schizophrenia and that its C allele increases dysbindin-1B mRNA expression. Overexpression of dysbindin-1B and its lack of the PEST domain contribute to aggresome formation. Dysbindin-1B is capable of recruiting dysbindin-1A protein to the protein aggregates and inhibits neurite outgrowth. Increased Dysbindin-1B expression may contribute to the neurodevelopmental defects in schizophrenia.

## Materials and Methods

### Subjects

A total of 500 unrelated patients with paranoid schizophrenia (260 males and 240 females, aged 35.85±0.31 years) and 500 unrelated healthy controls (264 males and 236 females, aged 35.90±0.36 years) were recruited according to institutional IRB and national guidelines. All subjects were of Chinese Han origin. These 500 patients were all diagnosed as having paranoid schizophrenia by at least two psychiatrists according to the Statistical Manual of Mental Disorders, 4th Edition (DSM-IV) criteria based on the Structured Clinical Interview for DSM-IV (SCID). All patients were at the first episode and drug free. The control subjects were recruited from local communities. Those with history of major psychiatric or neurological disorders, psychiatric treatments or drug abuse, or family history of psychiatric disorders were excluded. For real-time quantitative PCR, 98 cases (46 males and 43 females, aged 33.79±1.22 years) and 98 controls (49 males and 49 females, aged 33.41±0.68 years) were also recruited based on the above criteria.

### SNPs identification

The major difference in mRNA sequences among Dysbindin-1A, -1C and Dysbindin-1B is whether intron 9 and exon 10 are included. We designed a pair of primers to amplify a 2 071-bp DNA fragment, including upstream of exon 9, intron 9 and exon 10, and sequence this fragment in 20 paranoid schizophrenic patients and 20 healthy controls. Only 4 SNPs were detected in it and no SNP was found in exon 9 in our study.

### SNPs genotyping

Genomic DNA was extracted from peripheral blood leukocytes using the phenol–chloroform protocol. The SNPs were genotyped by PCR-based sequencing method. Primers 5′-AATAATAGTTGCCAAGGGTGAT-3′ (forward) and 5′-GCGCTCTCAGTTTACCGTC-3′ (reverse) were used for PCR amplification and the conditions used were as follows: 95 °C for 10 min, followed by 35 cycles of 95 °C for 30 s, 58 °C for 30 s and 72 °C for 130 s, and finally by extension at 72 °C for 10 min. The PCR products were sequenced bidirectionally using ABI 3700 DNA sequencer (Perkin-Elmer, Applied Biosystems, Foster City, CA, USA).

### Real-time quantitative PCR

Total RNA was extracted from peripheral blood leukocytes or COS7 cells using the Trizol–chloroform method. The purity and integrity of total RNA were evaluated by ultraviolet-spectrophotometry and gel electrophoresis. Genomic DNA was removed by treatment with RQ1 RNase-free DNase (Promega, Madison, WI, USA). DNase-treated RNA was added to a 50-μl reverse transcriptional reaction for synthesis of complementary DNA (cDNA) with random primers (TIANGENE, Beijing, China), M-MLV reverse transcriptase (Promega) and rRNasin ribonuclease inhibitor (Promega). Glyceraldehyde-3-phosphate dehydrogenase was used as an internal control. RNA from COS7 cells were transcribed in a 20-μl reaction system with random primers (TIANGENE). The *NEO* gene located in pcDNA3.1/myc-his(−)B was used as an internal control.

For cDNA from peripheral blood leukocytes RNA, dysbindin-1 mRNA expression was measured by relative quantitative analysis on the ABI PRISM 7500 real-time PCR system (Applied Biosystems). The primers for real-time PCR amplification listed in Supplementary Table S1 were previously reported by Tang *et al.* [48]. A 20-μl quantitative PCR (qPCR) reaction containing 2 μl of cDNA was performed in triplicates. The comparative 2^−ΔΔCt^ method was used to relatively quantify target transcripts. For cDNA from COS7 cells, Dysbindin-1A and -1B mRNA expression were measured on the instrument as mentioned above. The primers and Taqman probes were listed in Supplementary Table S2. A 1.5 μl of cDNA samples were used for a 20-μl qPCR reaction in triplicate. The comparative 2^−ΔCt^ method was used to relatively quantify target transcripts.

### Construction of transgenic mouse expressing human dysbindin-1B-myc in C57/BL6 background

The transgenic mouse expressing human dysbindin-1B-myc in C57/BL6 was contructed by Biocytogen (Beijing, China). A LoxP site was inserted into intron 5 whereas other elements were inserted in intron 6 in proper order (Figure 2C). We crossed the dysbindin-1B-myc transgenic mouse with CMV-Cre mouse (from Biocytogen) in the same background to knockout endogenous dysbindin-1 by removing exon 6. Meanwhile, the human dysbindin-1B is induced to express under the control of endogenous promoter of *Dtnbp1*. The offsprings were genotyped to ensure the deletion of exon6 and Neo cassette. The expression of dysbindin-1B in the brain was verified by western blot (anti-myc tag) (Figure 2D).

### Protein solubility assay, MG132 treatment and immunoblotting

Lipofectamine2000 (Invitrogen, Carlsbad, CA, USA) was applied to transfect cells with myc-tagged control, dysbindin-1A and -1B. Transfected cells were lysed with RIPA buffer composed of 50 mm Tris-HCl (pH 7.4), 150 mm NaCl, 1% NP-40, 0.25% Na-deoxycholate, 1 mm EDTA, with proteinase inhibitor cocktail, 1 mm Na_3_VO_4_ and 1 mm PMSF, at 48 h of transfection. After centrifugation of cell lysates at 13 300 r.p.m. for 15 min at 4 °C, the supernatants and pellets were collected for solubility analysis. For the MG132 treatment, 10 μm MG132 was added to the medium of transfected HEK293 cells that were then incubated for 12 h before cell were harvested. The soluble fraction was collected and subjected to immunoblotting using Myc-Tag (9B11) mouse mAb (Cell Signaling Technology Inc., Danvers, MA, USA), and anti-α-tubulin (Sigma, St Louis, MO, USA).

### Primary cortical neuron culture and neurite outgrowth assay

Wild-type C57BL/6J or sandy mice [38] were used for primary cortical neuronal culture. For sandy mice, at E15.5, mothers were euthanized. Cortices from embryos were dissected and dissociated after trypsin digestion. Cells from individual embryos were plated on poly-L-lysine-coated coverslips. The cortical neurons were cultured in Neurobasal medium with B27 supplement (Invitrogen) for 48 h before transfection with GFP and dysbindin-1B-GFP by Lipofectamine2000 (Invitrogen). Neurons were then fixed with 4% paraformaldehyde in PBS and immunostained with anti β-III tubulin antibody (Abcam, Cambridge, MA, USA). The longest neurites of individual neurons were measured by NeuronJ software (ImageJ, NIH, Bethesda, MD, USA) [92]. The average neurite length was expressed as mean±s.e. based on the measurements of ~50 cells. Three independent experiments were performed and data were analyzed by *t*-test.

### Study approval

All the recruited subjects gave written informed consents before sample collection for genetic analysis. This study was approved by the Ethnics Committee of the Chinese Academy of Medical Sciences and Peking Union Medical College. All animal procedures were approved by the Institutional Animal Care and Use Committee of Chinese Academy of Medical Sciences and Peking Union Medical College and conformed to the national and international guidelines.

## Figures and Tables

**Table 1:** Allelic association of the *DTNBP1* gene with paranoid schizophrenia

*Polymorphism*	*Allele*	*CTR,* n *(%)*	*SCZ,* n *(%)*	χ^*2*^	P*-value* a	*Odd-Rs (95% CI)*
rs117610176	T	947(0.95)	900(0.90)	15.87	6.78e-005^a^	1.99 (1.41–2.80)
	C	53(0.05)	100(0.10)			
rs742106	T	849(0.85)	843(0.84)	0.14	0.71	1.05 (0.82–1.34)
	C	151(0.15)	157(0.16)			
rs34782642	A	576(0.58)	599(0.60)	1.09	0.30	0.91 (0.76–1.09)
	T	424(0.42)	401(0.40)			

CTR, control group; SCZ, paranoid schizophrenia.

a The corrected *P*-value from 10 000 permutations was 0.0003.

**Table 2:** Genotypic association of the *DTNBP1* gene with paranoid schizophrenia

*Polymorphism*	*Allele*	*CTR,* n *(%)*	*SCZ,* n *(%)*	χ^*2*^	P*-value* a	*Odd-Rs (95% CI)*
rs117610176	TT	447 (0.89)	400 (0.80)	17.28	3.22e-005^a^	1 (1–1)
	TC	53 (0.11)	100 (0.20)			2.11 (1.47–3.02)
rs742106	TT	362 (0.72)	350 (0.7)	3.24	0.20	1 (1–1)
	TC	125 (0.25)	143 (0.29)	4		1.18 (0.89–1.57)
	CC	13 (0.03)	7 (0.01)			0.56 (0.22–1.41)
rs34782642	AA	165 (0.33)	185 (0.37)	1.80	0.41	1 (1–1)
	AT	246 (0.49)	229 (0.46)	0		0.83 (0.63–1.10)
	TT	89 (0.18)	86 (0.17)			0.86 (0.60–1.24)

CTR, control group; SCZ, paranoid schizophrenia.

a The corrected *P*-value from 10 000 permutations was 0.0003.
