# Supplementary material for: Increased dysbindin-1B isoform expression in schizophrenia and its propensity in aggresome formation
Source: Cell Discov. 2015 Nov 10;1:15032–. doi: 10.1038/celldisc.2015.32 (PMC4860834; doi:10.1038/celldisc.2015.32)
Supplement: Supplementary Information [file celldisc201532-s1.pdf]

Supplementary materials, methods, figure legends and tables

Constructs and cell lines

The ORFs (open reading frames) encoding human dysbindin-1A, -1B and -1C were cloned into the pEGFP-N1, pcDNA3.1/myc-his(-)B and pcDNA3.1-HA vector. Dysbindin-1AΔPEST and the fragment containing exon 9 - intron 9 - exon 10 were cloned into pcDNA3.1/myc-his(-)B vector. Dysbindin-1A and -1B deleted the two coiled-coil domains were cloned into pcDNA3.1/myc-his(-)B and pcDNA3.1-HA vector, respectively (Fig. S6A). The ORF encoding human snapin was cloned into pTriEx-4 vector. HEK293, HEK293T, COS1 and COS7 cells were cultured in DMEM containing 10% FBS. Fluorescent microscopy was performed within 24–72 hrs after transfection, while RNA was extracted at a 48-hour time point after transfection.

Immunofluorescent microscopy

To stain cells, they were fixed using 4% paraformaldehyde in PBS or cold methanol, incubated in the blocking solution (5% normal goat serum, 0.3% Triton X-100 in PBS) for 1 hr and then with the primary antibodies overnight at 4°C, followed by incubation with fluorescein-conjugated secondary antibodies. The aggresome reporter systems included monoclonal antibodies against γ-tubulin (Sigma), anti-α-subunit of 20S proteosome antibody (Calbiochem, San Diego, CA, USA) and vimentin (Santa Cruz Biotechnology Inc., Santa Cruz, CA, USA). To stain lysosome, COS1 cells were incubated with LysoTracker DND-99 (Invitrogen; 1:10,000 in cell culture medium) at 37°C for 30 min, followed by wash and fixation. Images were

taken using an Olympus FV1000 confocal microscope and analyzed with the FV10-ASW software. To work out the proportion of cells containing aggregates upon MG132 treatment, COS1 cells were transfected with GFP-tagged dysbindin-1A and -1B. At 36 hrs of transfection, 10 μM MG132 was added to the medium and incubated for another 4 hrs.

The His-pull-down assay

Cells were washed twice using cold PBS at 48 hrs after transfection, and then lysed in RIPA buffer on ice. Supernatants were collected after centrifugation at 13,300 rpm, 4°C for 15 min; 20-μg purified His-dysbindin-1A, Snapin-His or His-Smt3 (as negative control) protein was added to the supernatants and rotated at 4°C for 2 hrs; 20-μl 50% NTA resin slurry (prepared with wash buffer) was added, rotated at 4°C for 1 hr and then centrifuged at 10,000×g for 30 s. After washing, the proteins precipitated with the resin were dissolved and analyzed using western blotting.

DTSP crosslinking analysis

The cross-linker DTSP (2 mM, Sigma) or control was added to cells 48 hrs following transfection of COS1 cells with the control vector or plasmid expressing HA-tagged dysbindin-1A and stood for 2 hrs at 4°C. After cell lysis, an equal amount of total proteins from each group was used for immunoblotting.

The co-immunoprecipitation assay

HEK293 cells were washed twice using cold PBS at 48 hrs after transfection, and then lysed in RIPA buffer on ice. Supernatants were collected after centrifugation at 13,300 rpm, 4°C for 15 min; Protein-A-agrose beads (Roche) and anti-myc or

anti-HA antibody were added into the supernatants respectively and rotated at 4°C overnight. After washing, the immunoprecipitated samples were analyzed using western blotting.

Supplementary figure legends

Fig S1. Subcellular distribution of dysbindin-1A, -1B, -1C and dysbindin-1AΔPEST in cell lines

(A) GFP-tagged dysbindin-1B forms aggregates in transfected COS1 cells (arrow). (B) The proportion of aggregate-containing cells among those expressing GFP-tagged dysbindin-1A, -1B and -1C (dys1A-GFP, dys1B-GFP, dys1C-GFP) shown in panel A. (C) Myc-tagged dysbindin-1B forms aggregates in transfected COS1 cells (arrow). (D) The proportion of aggregate-containing cells among those expressing myc-tagged dysbidnin-1A, -1B and -1C (dys1A-myc, dys1B-myc, dys1C-myc) shown in panel C. (E) GFP-tagged dysbindin-1B forms aggregates in transfected HEK293 cells (arrow). (F) The proportion of cells containing aggregates among those expressing GFP-tagged dysbindin-1A, -1B or -1C in HEK293 cells. (G) Expression of dys1AΔPEST-myc deletion mutant in COS7 cells leads to aggregate formation (arrow). (H) Quantification of the percentage of cells containing aggregates in COS7 cells expressing myc-tagged dysbindin-1A, -1B and dysbindin-1AΔPEST. Data are presented as mean±SEM. *** $p<0.001$. ns, not significant. Scale bar: 20μm.

Fig S2. Formation of dysbindin-1A oligomers in cultured cells

COS1 cells transfected with the control vector or HA-tagged dysbindin-1A were treated with cross-linking agent DTSP. DMSO was added as the control vehicle. The left panel represents the formation of dysbindin-1A oligomers in a higher molecular

weight than its monomers. The right panel represents the monomers of dysbindin-1A without DTSP treatment.

Fig S3. Dysbindin-1A or -1B was not pulled down by purified His-Smt3

Purified His-Smt3 was used as a negative control in the pull-down assay. No HA-tagged dysbindin-1A or -1B were detected in the pull-down samples. Coomassie blue staining showed the purified His-Smt3 in the middle panel.

Fig S4. Two coiled-coil domains in dysbindin-1A and dysbindin-1B were required for their interactions.

(A) Schematic illustration of Myc-tagged dysbindin-1A and HA-tagged dysbindin-1B deleting the two coiled-coiled domains. (B) HEK293T cells co-transfected with Myc-tagged dysbindin-1A-ΔCCD and HA-tagged dysbindin-1B-ΔCCD were lysated and immunoprecipitated using anti-Myc antibody, followed by immunobloting as indicated. CCD, coiled-coil domain.

Fig S5. Dysbindin-1B was immunoprecipated with DISC1

HEK293T cells co-transfected with HA-tagged dysbindin-1B and Myc-tagged DISC1 were lysed and immunoprecipitated using anti-Myc antibody, followed by immunobloting as indicated.

Fig S6. Dysbindin-1B interacts with Snapin and recurite it to aggresome

(A) Transfected HEK293T cells were lysated and pull down by His-tagged Snapin. Both of HA-tagged dysbindin-1A and -1B were detected in the pull-down samples. Coomassie blue staining showed the His-tagged Snapin. (B) Immunostaining of COS1 cells co-transfected with HA-tagged Snapin and GFP-tagged dysbindin-1B. The arrow shows the co-localization of HA-tagged Snapin with GFP-tagged dysbindin-1B in the dysbindin-1B aggregate-containing cells at the perinuclear region. Scale bar: 20μm.

Supplementary Table 1. The real-time PCR primers specific for total *DTNBP1* mRNA, *DTNBP1b* only, *DTNBP1c* only and *GAPDH*.

Total *DTNBP1* mRNA	DYS-102-F	5'-CAGCAGGACATGGAGCAGTA-3'
	DYS-102-R	5'-CATGTCCACGTTCACTTCCA-3'
DTNBP1b	Dysbindin-1B-F	5'-ACGCATAAACCCCCAAGC-3'
	Dysbindin-1B-R	5'-GACGGAACCACACCACTGT-3'
DTNBP1c	DYS-1C-F	5'-AGTGAAAAGCAAACCCAGTTG-3'
	DYS-1C-R	5'-GTCCTGCCCAAAAGAAACAC-3'
GAPDH	GAPDH-RT-F	5'-ACTTCAACAGCGACACCCACT-3'
	GAPDH-RT-R	5'-GCCAAATTCGTTGTCATACCAG-3'

Supplementary Table 2. The real-time PCR primers and Taqman probe specific for *DTNBP1(a+c)* mRNA, *DTNBP1b* only and *NEO*.

DTNBP1(a+c)	DYS-E9E10-F	5'-GACATGCTGGAGCAGATGGA-3'
	DYS-E9E10-R	5'-GCAGGTGGAGGAAGAAGAAGGT-3'
	Taqman probe	5'-AGGGCCTGAATCCAGTAC-3'
DTNBP1b	DYS-B-F	5'-GTGGTGTGGTTCCGTCATGA-3'
	DYS-B-R	5'-AACGCCAGTCCTTAACCACAA-3'
	Taqman probe	5'-ACTCCGGTAACTTTG-3'
NEO	Neo-F	5'-CTTGGGTGGAGAGGCTATTCG-3'
	Neo-R	5'-GGACAGGTCGGTCTTGACAAA-3'
	Taqman probe	5'-CTGATGCCGCCGTGTT-3'
