# Supplementary figures and images for: Increased dysbindin-1B isoform expression in schizophrenia and its propensity in aggresome formation
Source: Cell Discov. 2015 Nov 10;1:15032–. doi: 10.1038/celldisc.2015.32 (PMC4860834; doi:10.1038/celldisc.2015.32)

Fig S1

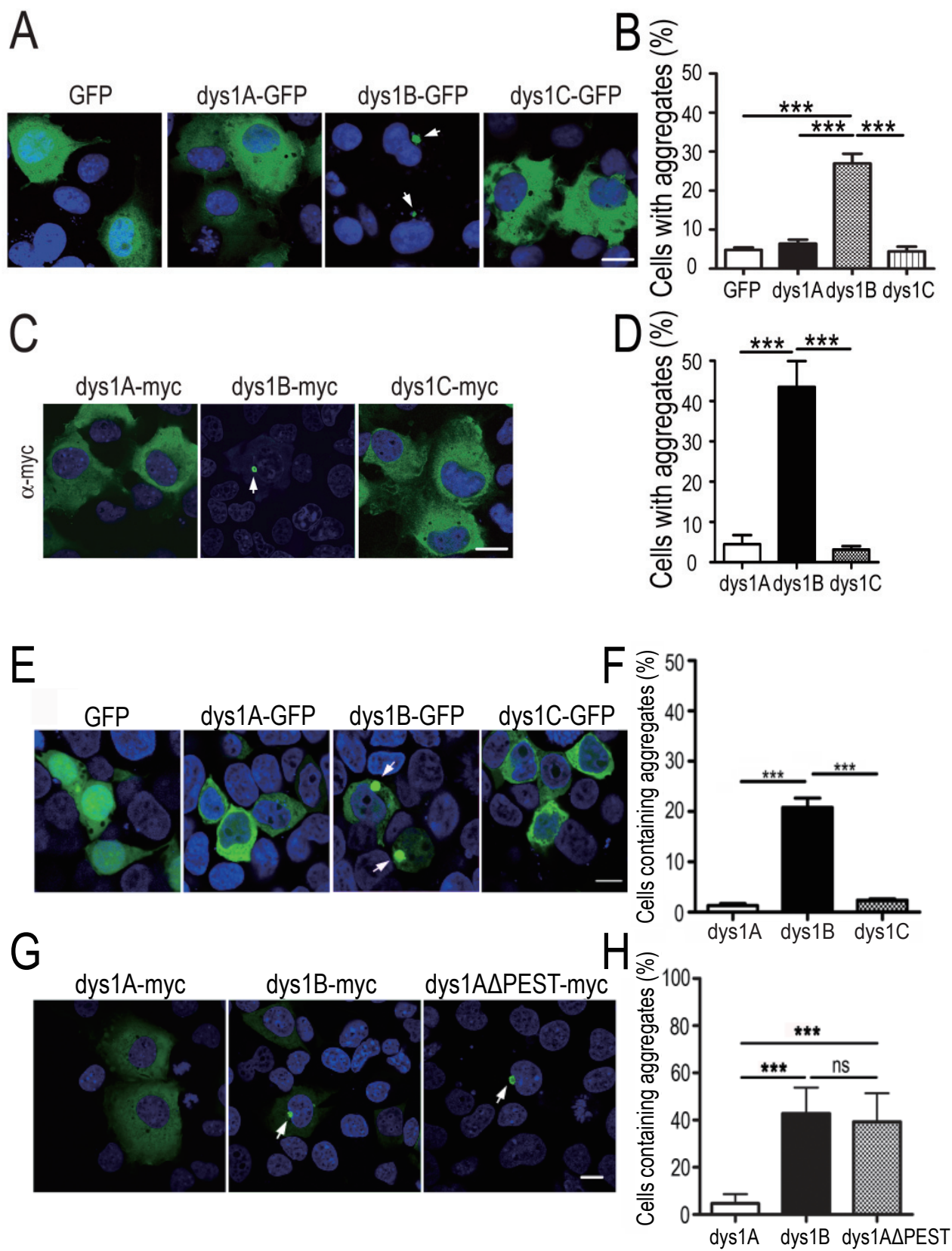

Supplement: Supplementary Figure S1 [file celldisc201532-s2.pdf]

Fig S2

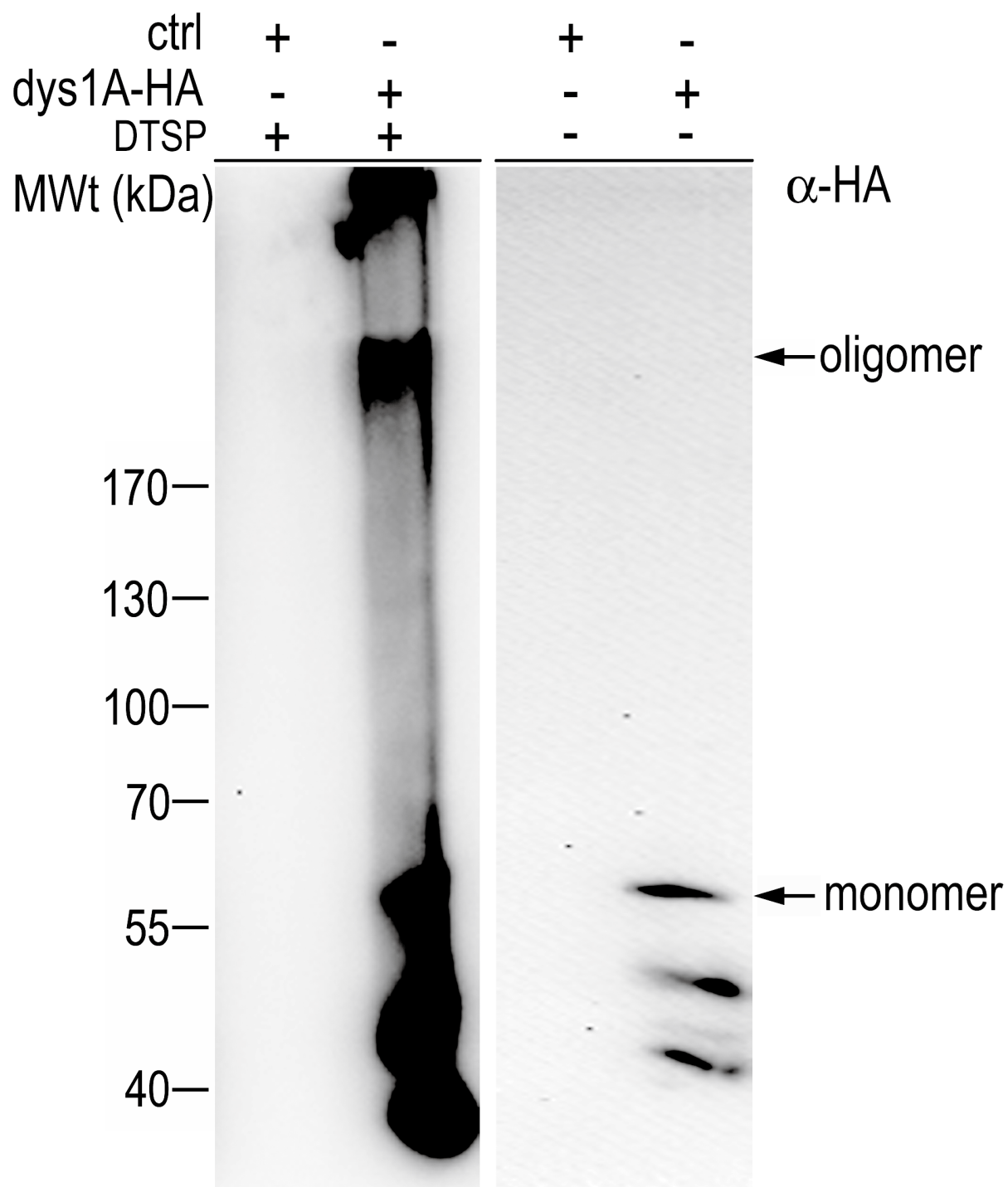

Supplement: Supplementary Figure S2 [file celldisc201532-s3.pdf]

Fig S3

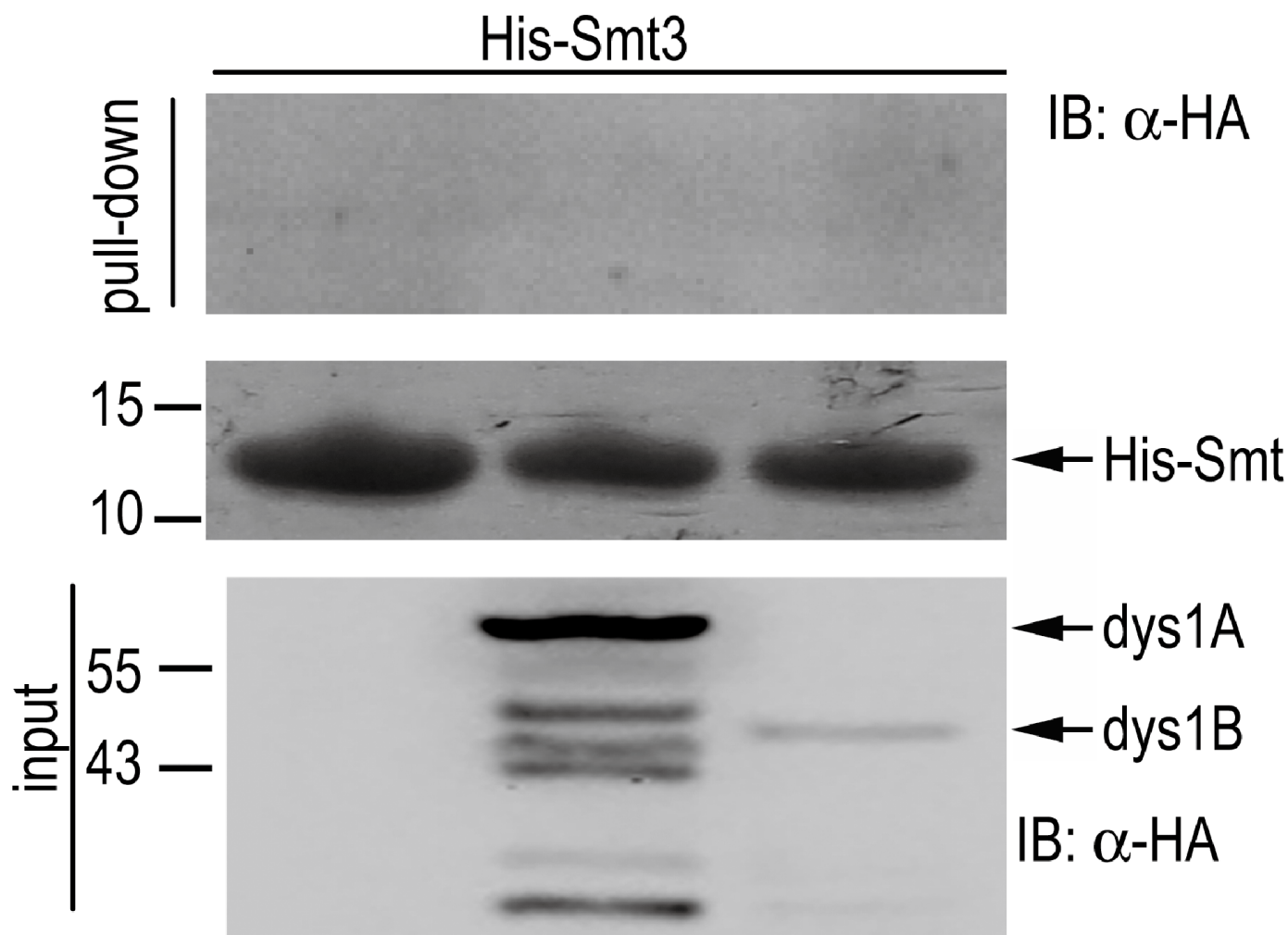

Supplement: Supplementary Figure S3 [file celldisc201532-s4.pdf]

Fig S4

A

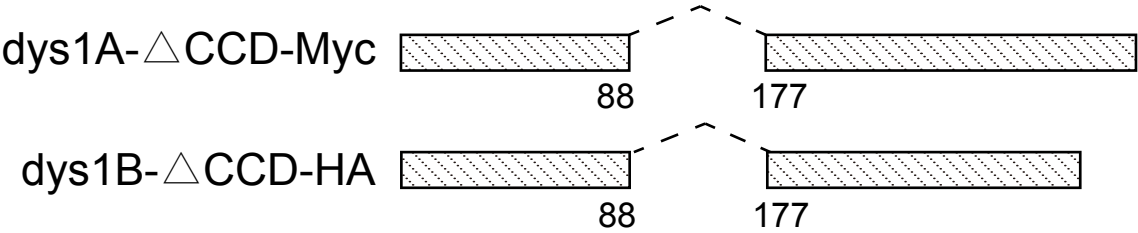

B

|                         |   |   |   |   |   |   |   |
|-------------------------|---|---|---|---|---|---|---|
| dys1A- $\Delta$ CCD-Myc | - | + | - | + | + | - | - |
| dys1B- $\Delta$ CCD-HA  | - | - | + | + | - | + | - |
| dys1A-Myc               | - | - | - | - | - | + | + |
| dys1B-HA                | - | - | - | - | + | - | + |

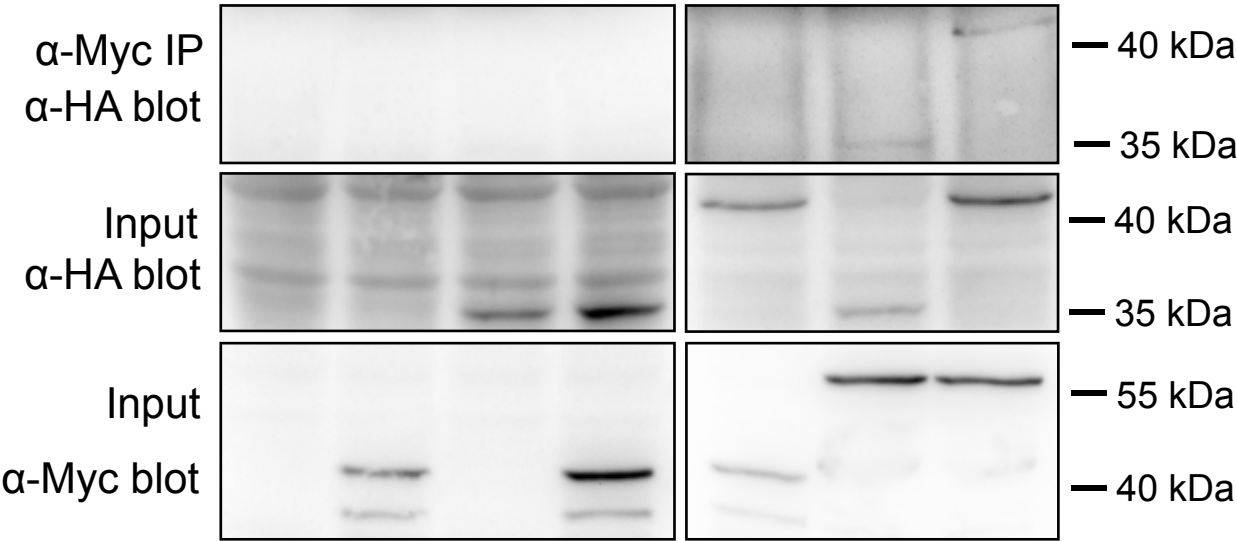

Supplement: Supplementary Figure S4 [file celldisc201532-s5.pdf]

Fig S5

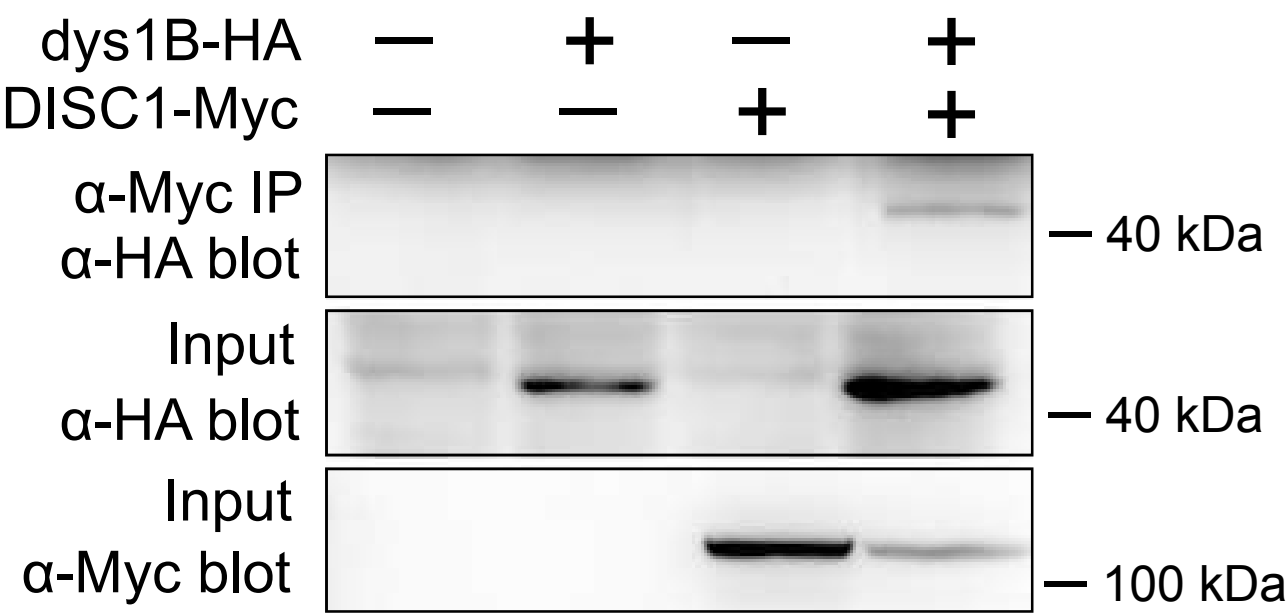

Supplement: Supplementary Figure S5 [file celldisc201532-s6.pdf]

Fig S6

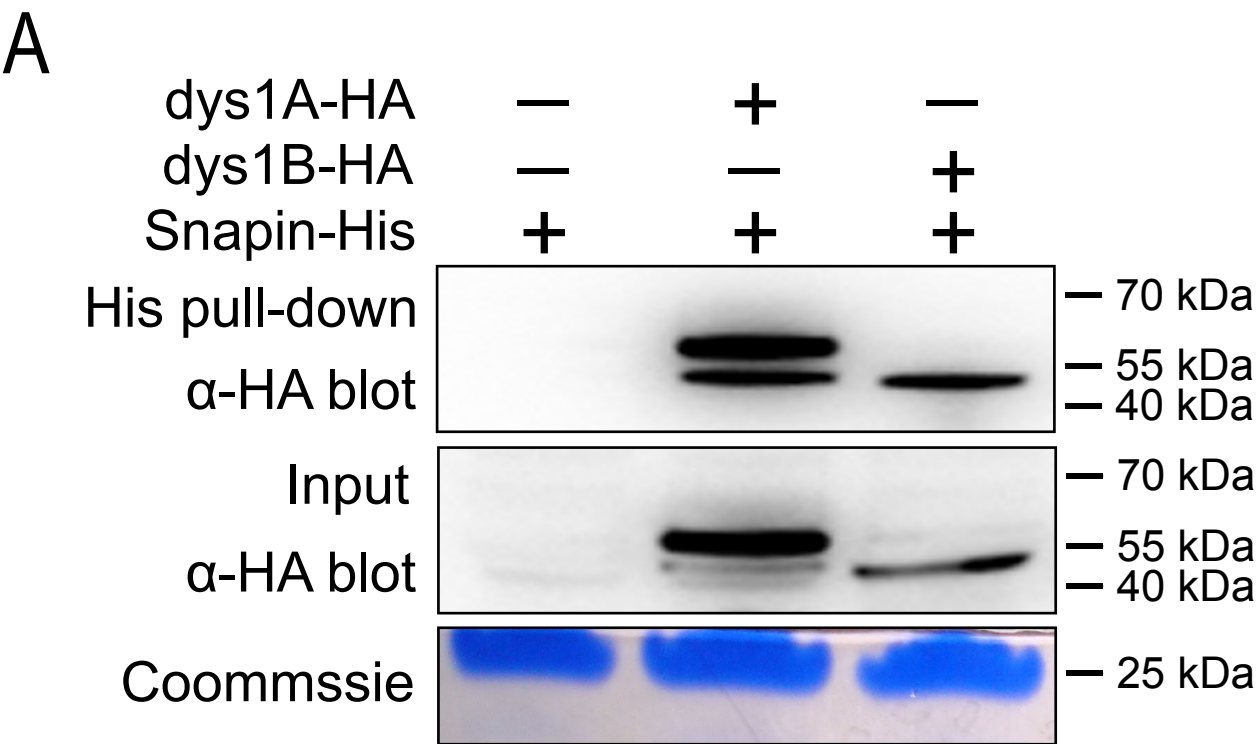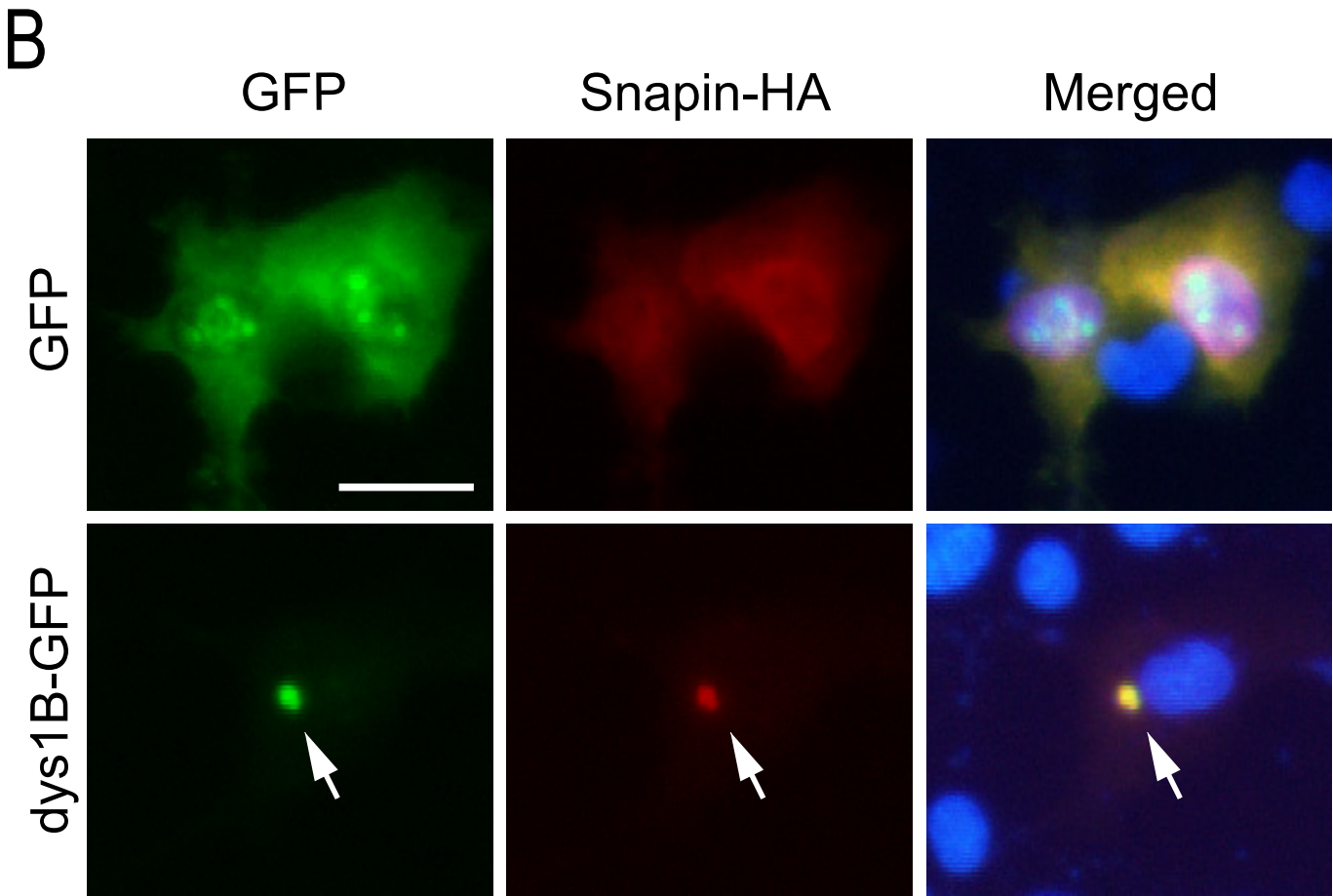

Supplement: Supplementary Figure S6 [file celldisc201532-s7.pdf]
